# #emergencymedicine: A TikTok Content Analysis of Emergency Medicine-related Content

**DOI:** 10.5811/westjem.19466

**Published:** 2024-11-27

**Authors:** Madison Stolly, Erika Wilt, Nathan Gembreska, Mohamad Nawras, Emily Moore, Kelly Walker, Rhonda Hercher, Mohamad Moussa

**Affiliations:** * University of Toledo College of Medicine and Life Sciences, Toledo, Ohio; † Department of Emergency Medicine, University of Toledo College of Medicine and Life Sciences, Toledo, Ohio

## Abstract

**Background:**

TikTok has rapidly become one of the most extensively downloaded and used social media platforms worldwide. Our focus on emergency medicine (EM)-related content on TikTok is to identify what specific video characteristics result in higher degrees of audience engagement, defined in this study as a total of video likes, comments, and shares.

**Methods:**

Five second-year medical students using newly created TikTok accounts independently downloaded the first 100 videos to appear using the hashtag #emergencymedicine. The videos were reviewed for 52 variables. We performed a multiple linear regression analysis to examine the relationship between the variables and video engagement.

**Results:**

Of the examined videos, 45.8% (222/484) were produced by physicians (MD or DO). Approximately half, 50.0% (242/484), had an educational component, while 55.4% (268/484) of videos were judged to have an entertainment component. Preliminary findings indicate that among TikTok videos featuring #emergencymedicine, a statistically significant positive correlation exists between video engagement and the presence of a healthcare identifier, (ie, individuals wearing white coats or scrubs). No significant correlation was observed between video engagement and video creators’ self-identification as a healthcare professional, use of entertainment, or use of education. A notable negative correlation was identified between video engagement and the inclusion of music.

**Conclusion:**

We identified qualities associated with negative and positive correlation with video engagement. For the 100 videos, only healthcare attire, such as wearing a white coat or scrubs, showed a significant positive correlation with engagement, while those with background music showed a negative correlation. Our study offers insight into how EM professionals can effectively use characteristics associated with higher engagement rates to relay information to a wider audience on TikTok.

Population Health Research Capsule What do we already know about this issue?

*TikTok is a rapidly growing social media platform. Healthcare information increasingly permeates online media.*

What was the research question?

*Which specific characteristics of TikTok videos lead to increased engagement (total likes, comments, and shares)?*

What was the major finding of the study?

*There was a positive correlation with engagement and healthcare identifiers such as white coats or scrubs, and a negative correlation with engagement and music, P < 0.05.*

How does this improve population health?

*Our study provides insights into how EM professionals can use engagement-driving features to effectively reach a broader audience on TikTok.*

## INTRODUCTION

In the past several decades, social media has become an integral part of society, with its prominence continuing to grow. Consequently, the public has started to use social media as a resource for obtaining medical information regarding one’s own health, details on a specific condition, how to develop healthy lifestyles, etc.^1^ Given the relative ease of searching for information online or on social media and the amount of information available, over 80% of people with internet access now seek healthcare information online.^2^ The world of healthcare information has grown to be a large presence in the online world, but as the modalities of online information continue to change and grow, the question remains how to effectively disseminate information on these various platforms. Recently, researchers for the Royal College of Physicians using education theory explored the risks and benefits of using social media for medical education and ascertained its use as positive and “here to stay.”^3^

TikTok has rapidly emerged as the fastest-growing social media platform currently in use. With availability in over 150 countries and over two billion downloads, it superseded Facebook, Twitter, and Instagram in 2018 and 2019 as the most frequently downloaded application.^4^ Currently, 12% of the global population uses the app monthly.^5^ TikTok operates on the premise of short-form video content where users can create and share videos, which typically last between 15–60 seconds, covering a wide range of topics, from entertainment and humor to education and creativity.^6^ The app’s algorithm employs a recommendation system that analyzes user behavior, such as the videos they like, share, and comment on, to curate a personalized “For You Page” (FYP).^7^ This FYP showcases content tailored to each user’s interests, ensuring a highly engaging experience as it constantly introduces users to new and relevant content from creators worldwide. TikTok also has a variety of creator tools, including filters, effects, and soundtracks, that allows creators to produce visually appealing and entertaining content in multiple forms. The platform’s emphasis on discoverability and “going viral” has led to the rapid rise of internet sensations and trends, making TikTok a dynamic and influential force in the world of social media.^8^ Limited research has been conducted on the potential role it will play in healthcare information.

While prior analyses of social media health campaigns primarily focused on Facebook or Twitter, younger generations have begun to use image- and video-based platforms such as Instagram, Snapchat, or TikTok.^9^ Very little data has been gathered pertaining to the effectiveness of engagement and accuracy of using these novel platforms to communicate health information to the general population. The use of TikTok has proven to be a source of some health information, such as sex education,^10^ but no studies have been directed toward emergency medicine (EM) specifically. A study directed toward the validity of TikTok videos about diabetes education found the videos to be of an acceptable level of accuracy.^11^ However, neither of these studies addressed what made an engaging video. A recent study by Kassamali et al^12^ found that videos containing on-screen text, music, and healthcare attire showed the highest rates of engagement on TikTok videos related to dermatology. Additionally, Barta et al investigated influencer content on TikTok, and their study suggested that humor, originality, quality, and quantity of videos correlated with increased rates of engagement.^13^

With nearly 72% of Americans using some form of social media,^14^ and with TikTok videos being so short and easy to view, TikTok is a potential vehicle for EM education especially in the acute setting. Recent research has explored the concept of microlearning, which involves breaking down information into small, manageable segments that allow learners to absorb knowledge at their own pace. Preliminary findings indicate that microlearning enhances both learning outcomes and long-term memory retention compared to traditional learning methods. Given the concise nature of TikTok videos, this platform could potentially be used to facilitate microlearning.^15^ While there is a growing presence of physicians using social media, very few use them as a public platform to make educational videos.^16^ With so many different variables in video characteristics, the question raised is whether certain videos created by healthcare content creators have a higher impact on the general public. Such information could allow for the use of TikTok to become an effective educational platform for learners and patients alike to gain valuable knowledge about medical-related matters in a timely manner. In this study we sought to investigate whether specific factors present in TikTok videos related to EM correlate with higher levels of likes, comments, and shares, thereby reinforcing strategies for enhancing engagement and disseminating content to the public.

## METHODS

### Data Search and Collection

Data used in this analysis was used for qualitative research and was collected on December 4, 2022, at 8 pm by five separate medical students using newly created TikTok accounts to minimize the variation due to the TikTok algorithm. Videos were downloaded on the same date and time to ensure the least amount of variability of content available. Each account searched “#emergencymedicine” and downloaded the first 100 videos that were listed. This created a sample size of 500 videos to analyze. Despite these being new accounts and having no prior interaction, there was variation among the videos that were downloaded. Videos were excluded from the sample if likes, comments, or shares were not available. This reduced the total sample size to 484 videos. These videos were saved independently for later review and record, and all videos were saved with the watermark including views, likes and shares which was located on the left side of the video, and username of the original content creator. Once they were downloaded, students reviewed the spreadsheet together and assessed several sample TikTok videos together to ensure each video would be analyzed similarly to reduce variability in data collection. Each student used an Excel spreadsheet (Microsoft Corp, Redmond, WA) to collect the required information; this was sent for statistical analysis once complete.

### Data Coding

For each video, 52 characteristics were recorded based on the methods used by Raber et al^17^ who studied TikTok content related to #mediterraneandiet. These included descriptive characteristics including upload date, number of likes, number of comments, number of shares, total length of video, and other hashtags included in the video description. Creator information was collected and recorded using information from the creator’s TikTok page or linked social media. Creator characteristics recorded included poster name, username, number of followers, account description or tagline, account type (private, sponsored, company), hyperlinks to other social media or storefronts, and any medical or professional credentials. After collection, the content of each TikTok was assessed separately and scored by the student using a numeric system with 0 representing “no” and 1 representing “yes.” General characteristics assessed included use of music, use of humor, text overlay, presence of infographics, presence of educational information, attempt to directly sell a product or service, and presence of any promotion for a service or company. All content was also rated based on inclusion of personal anecdotes, conspiracy theories, the creator having MD/DO credentials, or study/data usage.

Some categories did not fit into the above coding style and had distinct numeric scales such as main message of the video (educational, entertainment, other), video structure (original content, stitch, response to comment, or other), video style (talking to camera, role-playing, dancing, emergency department (ED) with no host, or other), and whether a specific acute or chronic disease was mentioned in the video. The final part of the coding for each video included assessing whether there was any educational or entertainment aspect to the video. Per the spreadsheet based on Raber et al’s methods, we defined educational videos as video that primarily contains a medically educational aspect, or content offers medical information, to include aspects specific to EM; this included but was not limited to defining what happens in the ED or defining a medical condition. We defined entertainment videos as including humor, making jokes about medicine, dancing, or a song. These were discussed with the raters ahead of time to prevent confusion. Raters then typed a brief description in the spreadsheet.

### Statistical Analysis

The data for each of the spreadsheets was combined in a master spreadsheet and analyzed using a multiple regression model with SPSS software version 28.0.1.1 (SPSS Statistics, IBM Corp, Armonk NY). We compared all using *P*-value <0.05. The independent variable for each video was the sum of likes, comments, and shares recorded in the spreadsheet, referred to as the average total engagement for that video. The dependent variables included in this analysis were the presence of background music, video creator attire, identification as a healthcare professional, account designation of healthcare professional, message of the video (education or entertainment), humor, and the presence of text overlays.

## RESULTS

Results of the average engagement (ie, total likes, comments, and shares) are shown in the Table and Figure. These were separated into the presence or absence of healthcare attire, entertainment, education, music, in-video credentials, humor, account designation of MD/DO, and text overlay for further evaluation through linear regression analysis.

An elevated level of video engagement exhibited a direct positive correlation with videos featuring creators wearing healthcare identifiers such as a white coat or scrubs, with a statistically significant *P*-value of less than 0.02. Moreover, the analysis indicated a decrease in video engagement when background music was present, with a *P*-value of less than 0.03. Conversely, there was a lack of compelling evidence supporting the notion that indicators such as the creator’s age, explicit self-identification as a healthcare professional within the video, or the account’s designation as a healthcare-related profile had a significant impact on overall video engagement. Furthermore, no statistically significant correlations were observed between an increased amount of engagement and various other factors including the presence of education, entertainment, humor, or the presence of text overlays.

**Figure.**
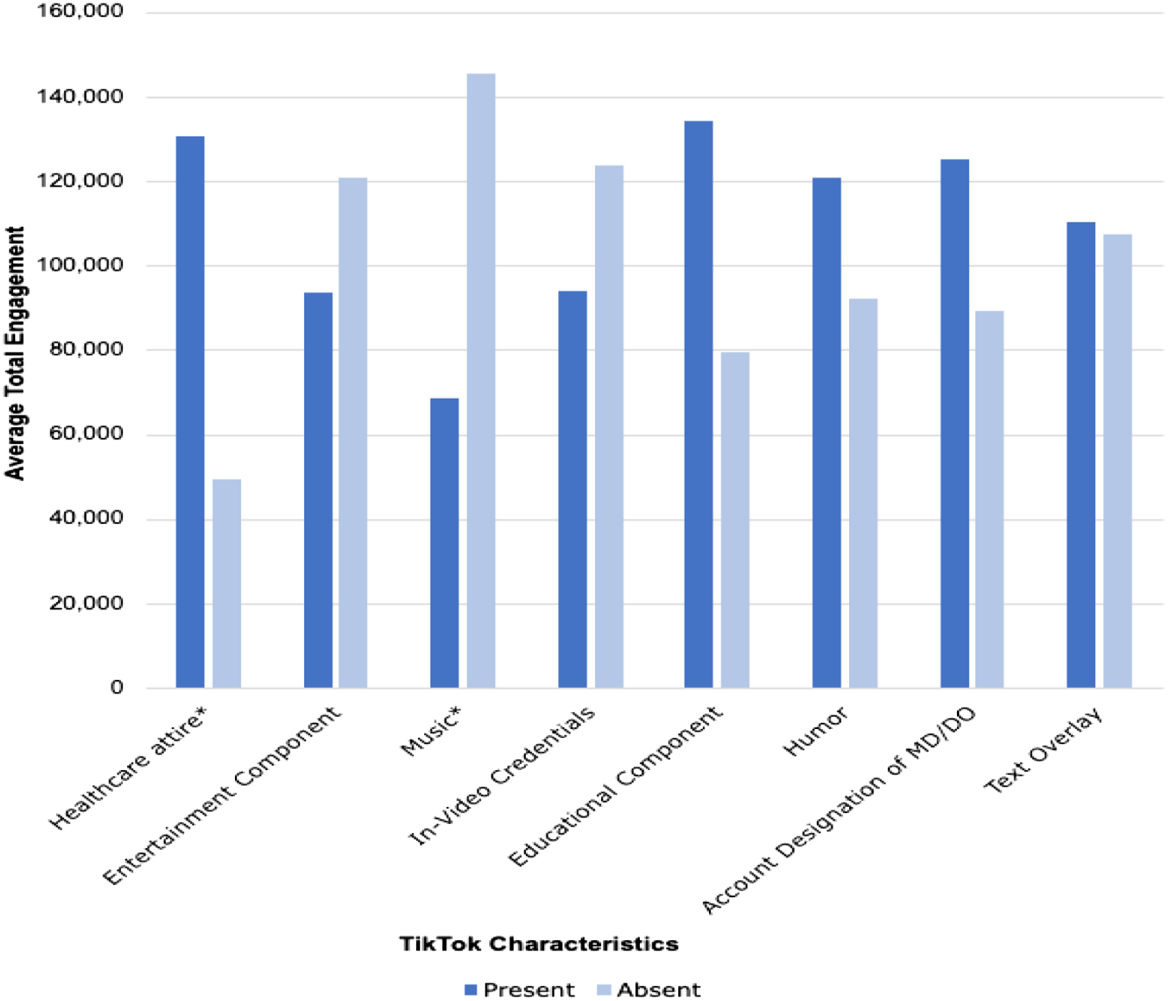
Total Average engagement of TikTok content based on the presence or absence of specific video characteristics.

**Table.** Average engagement levels (likes, comments, shares) of TikTok users based on various video characteristics.

	Present	Absent
Healthcare attire*	130,725.00	49,262.05
Entertainment component	93,574.84	120,774.50
Music^**^	68,745.16	145,558.08
In-video credentials	93,988.79	123,699.12
Humor	120,769.56	92,047.63
Account designation of MD/DO	125,201.33	89,126.45
Text overlay	110,236.07	107,255.79

* Positive correlation with creators wearing white coats/scrubs.

** Negative correlation with background music.

## DISCUSSION

As in the field of medicine, the landscape of social media is in a constant state of evolution, continuously introducing and expanding novel information distribution methods. This study has provided valuable insights into the utility of TikTok and its effective application in increasing engagement in videos pertaining to EM. By creating new accounts, the investigation tried to mimic a FYP free of healthcare content bias to further understand what a general user may see on their page by searching the hashtag, #emergencymedicine. Furthermore, the research delved into the strategies available to emergency healthcare professionals for leveraging TikTok as a medium to disseminate educational content to the general public.

Interestingly, it was discovered that the primary factor influencing the engagement of a TikTok video is neither the credentials nor the message of the content, but rather the attire of the video creator. There was no benefit to the creator stating whether they were a licensed professional. Further, there was no discernable inclination toward either educational or entertaining content. This suggests that users are openly receptive to digesting educational and entertaining medical content on the platform. This offers guidance on how to engage effectively with the broader audience in the medical domain through utilization of the FYP. It also underscores the unique nature of contemporary social media platforms, where rapid, split-second judgments play a critical role, emphasizing the need to capture viewers’ attention swiftly to achieve engagement.

The level of engagement of TikTok videos may prove to be content dependent on which variables matter most in levels of engagement. For instance, Barta et al discovered that incorporating humor was advantageous in creating videos to be a successful influencer.^13^ However, when assessing engagement specifically within the subject matter of EM, humor did not demonstrate any beneficial impact when added to the videos. Our findings further support the study done in 2021 by Kassamali et al as they investigated the “virality” of TikToks relating to dermatology education videos and found that medical attire was positively correlated with overall virality.^12^ Additionally, the study suggested increased engagement with videos that contained music in the TikTok video.

Conversely, the findings in this study suggest that TikTok videos related to EM with background music acted as deterrents for viewers, implying educators might achieve greater effectiveness by speaking directly to the camera. Consequently, some aspects of TikTok videos may be content-dependent even within the healthcare field. One pioneering aspect of social media is the ability to connect ideas. Aside from content creators accessing their intended audiences, it also allows for content creators to connect with one another, collaborating for the best outcome. Thus, creators can share ideas not only regarding their content but also how to best engage general audiences.

According to recent data, 42% of all users are between the ages of 18–24, followed by users between the ages of 13–17 (27%).^8^ In light of the continued surge in popularity of this social media platform, it becomes increasingly imperative to refine approaches aimed at effectively engaging TikTok’s diverse audience. Given that the majority of TikTok users fall within the age range of 13–24, it presents a distinctive and valuable opportunity to connect with this demographic in the realm of health engagement. Using a widely adopted communication medium, EM professionals could effectively employ this platform to disseminate instructive information to the population. This research has opened the door to many of the possible merits of social media in medical academia and outreach. This research has demonstrated what elements to incorporate and avoid when creating content with the goal to encourage engagement. It has illustrated which characteristics of content within EM are most likely to draw significant engagement. Notably, wearing healthcare attire appears to enhance the likelihood of the public encountering the video. This proved to be more advantageous compared to other factors presumed to be influential such as explicitly stating one’s healthcare profession, incorporating entertainment elements, or displaying professional credentials such as MD or DO next to one’s name.

Our research opens the door for many new avenues of the intersection of social media and medicine, specifically medical content and how general public users can interact with it. Further analysis could be determined to see what makes social media videos not only engaging but effective teaching tools. Other potential research could include assessing the medical content of other social media sites such as Instagram and Twitter, given their large presence. Other metrics of medical videos on social media could be analyzed as well, including an exploration of the user demographics that engage with various types of EM content. Additionally, this could include video accuracy, potentially looking at more specific medical conditions, procedures, or quality improvement. Generally, our research goal is to reach out to a wide group of people, supporting accurate EM content and encouraging active participation in a technology-centered world.

## LIMITATIONS

For this research study we engaged five autonomous assessors, each equipped with newly established accounts, to evaluate content simultaneously on a specific day. The aim was to manipulate an impartial search, recognizing that the TikTok algorithm dynamically alters the content presented to viewers, contingent on their previously indicated preferences and the prevailing time of the year. By employing this methodology, we sought to mitigate the influence of personalized user history and seasonal variations, thereby facilitating a more unbiased assessment of the platform’s content dissemination mechanisms. Nonetheless, the study encountered certain limitations.

Due to feasibility purposes, the study relied on medical students to collect the data. The reliance on a relatively small cohort of only five assessors, whose medical education level was at the second or third year, may have imposed constraints on their ability to accurately interpret the information presented, given their training had yet to be fully comprehensive and specific to the ED domain. Further, there was not an analysis performed on the raters’ inter-rater reliability in performing the analysis of the videos. Secondly, the evaluation of merely 100 videos per assessor raises the possibility of potential overlap in the video samples observed, which could inadvertently have affected the diversity of the data pool. The study also reviewed only videos that used #emergencymedicine, assuming the content users appropriately used the hashtag for content relevant to EM, and that it is the preferred hashtag used. Lastly, an inherent in-group bias might have been introduced, as the assessors may have tended to perceive the videos as targeted toward other healthcare professionals, despite their intended audience being the public. These limitations warrant careful consideration when interpreting the findings and underscore the necessity for prudently addressing them in future research in this domain.

## CONCLUSION

TikTok is a platform that uses videos to relay messages in a restricted amount of time, emphasizing how important it is for content creators to use time and video characteristics wisely to engage the audience before their attention may be diverted elsewhere. This suggests videos containing a quality that significantly increases total engagement may be used to share information to a greater audience. In addition, avoiding characteristics associated with negative engagement can contribute to reaching a wider audience. Based on our findings, wearing healthcare-related attire, such as a white coat or scrubs, showed a statistically significant increase in total engagement. Conversely, background music in the videos decreased the total engagement. We believe these results suggest EM professionals can create videos using certain approaches, such as wearing healthcare attire, to share healthcare information to a greater number of individuals on the TikTok platform. While it may not be immediately clear how TikTok can be used to disseminate healthcare information, our findings provide some much-needed insight into the current relationship between TikTok and #emergency medicine.
